# Drop–Dry Deposition of Ni(OH)_2_ Precursors for Fabrication of NiO Thin Films

**DOI:** 10.3390/ma15134513

**Published:** 2022-06-27

**Authors:** Tong Li, Tetsuya Okada, Masaya Ichimura

**Affiliations:** Department of Electrical and Mechanical Engineering, Nagoya Institute of Technology, Gokiso, Showa, Nagoya 466-8555, Japan; t.li.463@stn.nitech.ac.jp (T.L.); 34413042@stn.nitech.ac.jp (T.O.)

**Keywords:** drop–dry deposition, Ni(OH)_2_, NiO, heterojunction

## Abstract

Drop–dry deposition (DDD) is a method of depositing thin films by heating and drying the deposition solution dropped on a substrate. We prepared Ni(OH)_2_ precursor thin films by DDD and annealed them in air to prepare NiO thin films. The appropriate deposition conditions were found by changing the number of drop–dry cycles and the concentrations of chemicals in the solution, and the Ni(OH)_2_ precursor film with a thickness of 0.3 μm and optical transmittance of more than 95% was successfully deposited. Raman and X-ray diffraction measurements were performed, and it was found that the NiO film was successfully fabricated after annealing at 400 °C. The p-type conductivity of the annealed film was confirmed by photoelectrochemical measurements. In addition, we prepared n-type ZnO by electrochemical deposition on NiO thin films. The current–voltage measurement results show that the ZnO/NiO heterojunction had rectification properties.

## 1. Introduction

Metal oxides (MOs) have a wide distribution of bandgaps covering wavelengths from infrared to ultraviolet, and constituent elements of most of them are abundant in the earth crust. Several MOs have been widely used in sensors, transparent electrodes and solar cells, such as TiO_2_, SnO_2_, In_2_O_3_ and ZnO. Those MOs are inherently n-type due to oxygen vacancies.

Among MOs, nickel oxide NiO is an environment-friendly p-type semiconductor material with a wide direct bandgap (Eg = 3.2–3.8 eV) [1]. Wide bandgap materials with inherent p-type conductivity are relatively rare [2,3], and NiO can be regarded as the most popular one. Transparent solar cells and photodetectors based on NiO/ZnO heterostructures have been studied [4,5,6,7,8,9,10,11,12,13]. NiO can form heterojunctions with a variety of other materials, such as Al_2_O_3_, Fe_2_O_3_, Ga_2_O_3_, GaN and organic semiconductors, and can be applied to water splitting [14,15,16], diode [17,18], sensor [19,20] and dye-sensitized photocathodes [21,22]. NiO layers can also be used as a transparent hole transport layer of perovskite solar cells [23] and in the edge termination of Ga_2_O_3_ Schottky diodes [24].

NiO films have been fabricated by various deposition techniques, which include physical vapor deposition [25,26], magnetron sputtering [27,28,29], spray pyrolysis [30,31,32], sol–gel technique [33,34,35,36], electrochemical deposition (ECD) method [37,38,39,40] and chemical bath deposition (CBD) [41]. While NiO can be directly deposited in a vacuum or at high temperatures, in the chemical methods such as ECD and CBD, Ni(OH)_2_ was first deposited and converted to NiO by annealing. For example, Ivanova et al. deposited Ni(OH)_2_ by the sol–gel technique using nickel acetate (Ni(CH_3_COO)_2_·4H_2_O) dissolved in absolute ethanol, and annealed it in air at different temperatures (200–500 °C) to fabricate a NiO thin film [36]. Nam and Kim deposited Ni(OH)_2_ by ECD using Ni(NO_3_)_2_ and annealed it in air at 300 °C to prepare a NiO thin film for supercapacitor applications [40]. Liu et al. deposited Ni(OH)_2_ by CBD using a Ni(NO_3_)_2_, ethanol and urea solution, and annealed it in air at 400 °C to prepare a NiO/ZnO heterojunction with high photocatalytic activity [41]. Ni(OH)_2_ itself has also been widely investigated for applications as catalyst, electrode and electrochromic materials [42].

**Figure 1:**
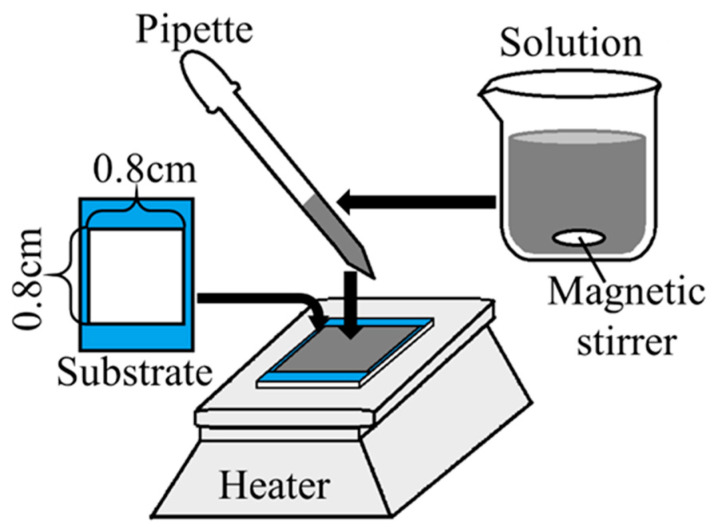
Apparatus of the drop–dry deposition method.

In this paper, Ni(OH)_2_ precursors are deposited by the drop–dry deposition (DDD) method and NiO thin films are fabricated by the annealing process. Figure 1 shows the schematic of DDD. DDD is a method of depositing a thin film by dropping and drying a solution on the substrate. DDD only uses a heating plate: the equipment required is simple and inexpensive. In addition, material efficiencies are high compared with other chemical techniques. Magnesium hydroxide and cobalt hydroxide have been successfully fabricated by DDD [43,44], but it has never been applied for nickel hydroxide. Owing to its simplicity and low cost, the fabrication of NiO thin films by DDD and annealing will be advantageous for various applications. In this work, we also fabricated ZnO/NiO heterojunction diodes using ECD-prepared ZnO. As shown below, rectification was confirmed, which indicates that DDD can be used in the fabrication of semiconductor devices based on NiO.

## 2. Experimental Section

The deposition solution for the Ni(OH)_2_ precursor was prepared by dissolving nickel(II) nitrate hexahydrate (Ni(NO_3_)_2_·6H_2_O, minimum 98% purity, Kanto Chemical Co. Inc., Tokyo, Japan) and sodium hydroxide (NaOH, minimum 97% purity, Kanto Chemical Co. Inc., Tokyo, Japan) in pure water (with a specific resistance of 18.2 MΩcm), and stirring with a magnetic stirrer. The indium–tin–oxide (ITO)-coated glass substrate (1.0 × 3.0 cm^2^, 10 Ω/sq) was degreased with acetone and washed with pure water, and the deposition area was limited to 0.8 × 0.8 cm^2^ by masking tape (adhesive strength 0.6 N/20 mm). The masking tape was peeled off after the deposition. A pipette was used to drop 0.05 mL of the solution onto the deposition area each time. The substrate was placed on the heater plate and heated, and the temperature of the heater plate was monitored to maintain it at 60 °C until it was visually observed that water on the substrate was evaporated completely. Next, the substrate was rinsed with pure water and blown with nitrogen gas. The Ni(OH)_2_ film was deposited by repeating the above steps.

During the drying process (with evaporation of the solvent), the low solubility material first precipitates and is deposited on the substrate, and, then, the high solubility material precipitates. The stability of Ni(OH)_2_ in water has been investigated [45]. The hydrolysis and condensation reactions are not expected in the aqueous solution, and, thus, gel is not formed. The solubility of Ni(OH)_2_ is approximately 10^−6^ M when pH = 12 (NaOH concentration of 10 mM). Thus, with evaporation of water, Ni(OH)_2_ readily precipitates and is deposited on ITO. Then, NaNO_3_ precipitates on Ni(OH)_2_ and is washed away in the rinsing process. The overall reactions can be expressed as follows.

Ni(NO_3_)_2_ + 2NaOH = Ni(OH)_2_ + 2NaNO_3_ (1)

The following conditions are examined to optimize the deposition process:

series (a): Number of drop–dry cycles: 2, 3, 4, 5 (Ni(NO_3_)_2_: 10 mM, NaOH: 15 mM).
series (b): Ni(NO_3_)_2_ concentrations: 5, 10, 15, 20 mM (NaOH: 10 mM, cycles: 2).
series (c): NaOH concentrations: 10, 15, 17.5, 20 mM (Ni(NO_3_)_2_: 10 mM, cycles: 2).

After depositing the thin film, the sample was placed in a tube furnace made of quartz, and an annealing treatment was performed at 400 °C for 1 h in air, resulting in thermal decomposition of Ni(OH)_2_ to NiO [39].

Ni(OH)_2_ → NiO + H_2_O (2)

**Figure 2:**
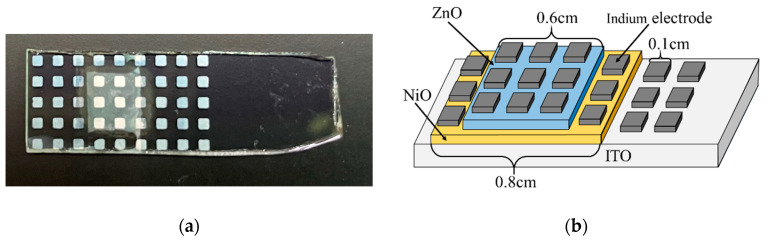
ZnO/NiO heterojunction: (**a**) photograph; (**b**) schematic diagram.

Figure 2 shows the schematic of the ZnO/NiO heterojunction. The ZnO/NiO heterojunction was fabricated by the following process. First, the NiO film with an area of 0.8 × 0.8 cm^2^ was fabricated on the ITO substrate by DDD and annealing. Then, a ZnO film was deposited on it by ECD. The deposition area of ZnO was limited to 0.6 × 0.6 cm^2^ by masking tape placed on the NiO film. The tape was removed after the deposition. The ZnO deposition solution contained 0.1 M zinc nitrate hexahydrate (Zn(NO_3_)_2_·6H_2_O, minimum 99% purity, Kanto Chemical Co. Inc., Tokyo, Japan) and was heated to 60 °C. The deposition time was 10 min, and the current density was −1.5 mA/cm^2^; the chemical reactions of the deposition are shown in ref. [46]. For the current-density–voltage (J-V) characterization, indium electrodes (0.1 × 0.1 cm^2^) were fabricated on the heterojunction by vacuum evaporation.

The thickness profiles were measured by an Accretech Surfcom-1400G profilometer. Optical transmittance was measured using a Jasco V-570 UV/VIS/NIR spectrometer. The transmittance data were obtained by dividing the transmittance of the sample by the reference data obtained for the ITO substrate without any deposits. Scanning electron microscope (SEM) images and Auger electron spectroscopy (AES) data were acquired using the JEOL JAMP-9500F field emission microprobe at a probe voltage of 10 keV. Raman spectroscopy measurement was performed using the Jasco NRS-3300 Raman spectroscope with excitation laser wavelength of 532 nm. X-ray diffraction (XRD) data were obtained using the SmartLab SE X-ray diffractometer (Rigaku) with a Cu Kα radiation source. Photoelectrochemical (PEC) measurement was carried out in a three-electrode system with a Ag/AgCl reference electrode. As the electrolyte, 100 mM sodium sulfate (Na_2_SO_4_) solution was used. For optical excitation, 100 mW/cm^2^ light from an ABET technologies 10500 solar simulator was irradiated intermittently at 5 s intervals, and the sample potential was swept within a range from −1 to 0 V and 0 to 1 V with a scanning rate of 5 mV/s.

## 3. Results and Discussion

### 3.1. Deposition Conditions

**Figure 3:**
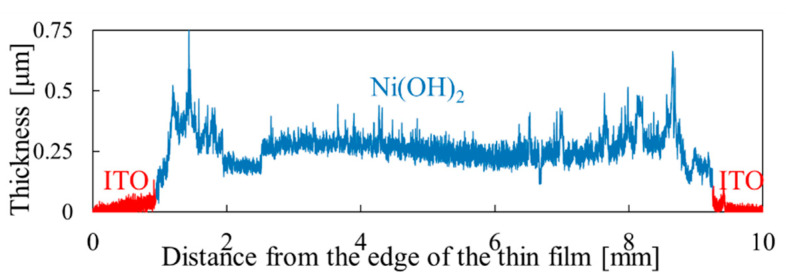
Thickness profile measurement results for the Ni(OH)_2_ thin film. (Ni(NO_3_)_2_ 10 mM, NaOH 15 mM, two deposition cycles).

**Figure 4:**
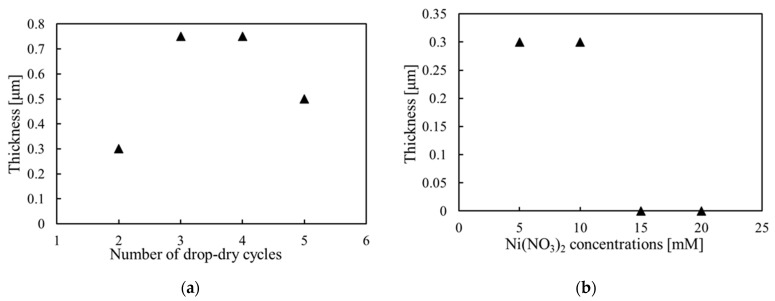

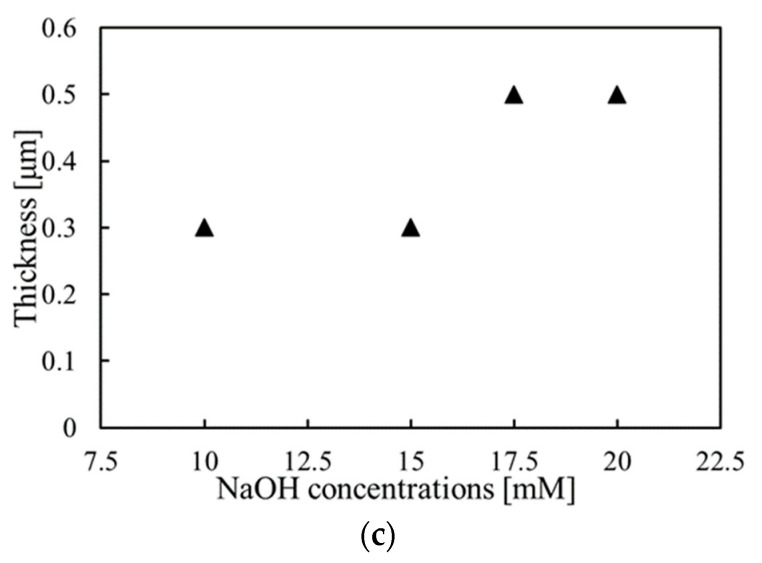
Film thickness under different deposition conditions: (**a**) change in the number of depositions; (**b**) change in Ni(NO_3_)_2_ concentration; (**c**) change in NaOH concentration.

Figure 3 shows an example of the thickness profile obtained to analyze the film thickness and roughness. Figure 4 shows the thicknesses of the samples deposited under different deposition conditions. Figure 4a shows the dependence of the film thickness on the cycle numbers (series (a)). It can be seen from the figure that the film thickness increases from 0.3 μm to 0.75 μm when the number of drop–dry cycles changes from two to three. However, the thickness did not continue to increase after the fourth cycle, and the surface of the sample became hazy, with an increasing nonuniformity. When the cycle number was increased to five, the film cracked and partly peeled off, and the thickness decreased instead. This shows that the thickness of the Ni(OH)_2_ films obtained by DDD is limited to below 0.75 μm.

Figure 4b shows the film thickness obtained with different Ni(NO_3_)_2_ concentrations (series (b)). With the increase in Ni(NO_3_)_2_ concentration (5–20 mM), the ratio of Ni(NO_3_)_2_ to NaOH increased from 1/2 to 2. The thicknesses of the samples obtained with 5 and 10 mM Ni(NO_3_)_2_ (the ratios of 1/2 and 1) were approximately 0.3 μm, but the surface was slightly rough for 10 mM Ni(NO_3_)_2_. The films were not successfully deposited with 15 and 20 mM Ni(NO_3_)_2_ (the ratios of 3/2 and 2), indicating that the Ni(NO_3_)_2_-to-NaOH ratio should not be higher than 3/2.

Figure 4c shows the film thickness obtained with different NaOH concentrations (series (c)). The thickness of the samples deposited with 10 and 15 mM NaOH (the Ni(NO_3_)_2_-to-NaOH ratio of 1 and 2/3) was approximately 0.3 μm. The sample thickness increased to 0.5 μm for 20 mM NaOH (the ratio of 1/2), but the sample surface became rough. This may be because aggregated Ni(OH)_2_ particles were formed in the solution and attached to the film surface. This indicates that the Ni(NO_3_)_2_ concentration ratio should be larger than 1/2.

**Figure 5:**
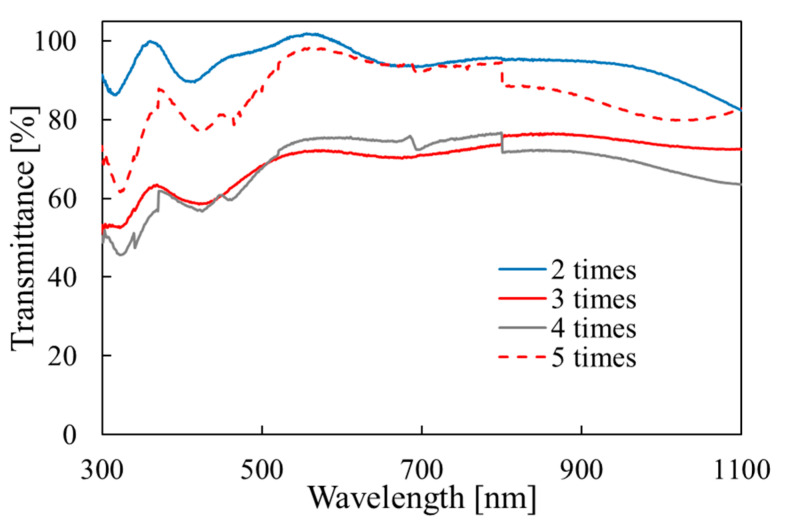
Optical transmittance for the samples fabricated with different deposition cycle numbers.

The transmittance results are shown in Figure 5 for different deposition cycle numbers. There is no absorption edge in the wavelength range in the figure. With the increase in the cycles from two to four, the film thickness increased from 0.3 to 0.75 μm as shown in Figure 4a, and the transmittance decreased from 90% to 70% in the visible region as shown in Figure 5. The thickness of the sample obtained with five cycles decreased as noted above, and the sample had a high transmittance (>90%) in the visible region. Thus, as the thickness increases, the surface roughness tends to increase and the transmittance tends to decrease. For the other deposition conditions (series (b) and (c)), the transmittance was in a range from 80 to 100%, and it tends to be larger for the thinner samples.

In the following characterization, we adopt the deposition condition: 10 mM Ni(NO_3_)_2_, 15 mM NaOH and two deposition cycles. Under this condition, the film thickness was approximately 0.3 μm, and the transmittance in the visible light region was 95% or more. In this optimized condition, the ratio of Ni(NO_3_)_2_ to NaOH is 2/3, not the stoichiometric ratio of 1/2. When the ratio is 1/2, Ni(OH)_2_ particle aggregation occurred in the solution, which affects the uniformity of the film. On the other hand, when the concentration ratio of Ni(NO_3_)_2_ exceeds 3/2, the deposition of the film failed. Thus, the ratio should be between 1/2 and 3/2, and the ratio of 2/3 was actually selected.

### 3.2. Characterization of As-Deposited and Annealed Films

By annealing at 400 °C, the thickness was reduced from 0.3 μm to approximately 0.12 μm. This is due to the conversion of Ni(OH)_2_ to NiO. The theoretical volume change can be calculated using the densities and molar masses of NiO and Ni(OH)_2_; with the same amount of Ni, the volume of NiO was expected to be 0.48 times that of Ni(OH)_2_, which is consistent with the experimental results.

**Figure 6:**
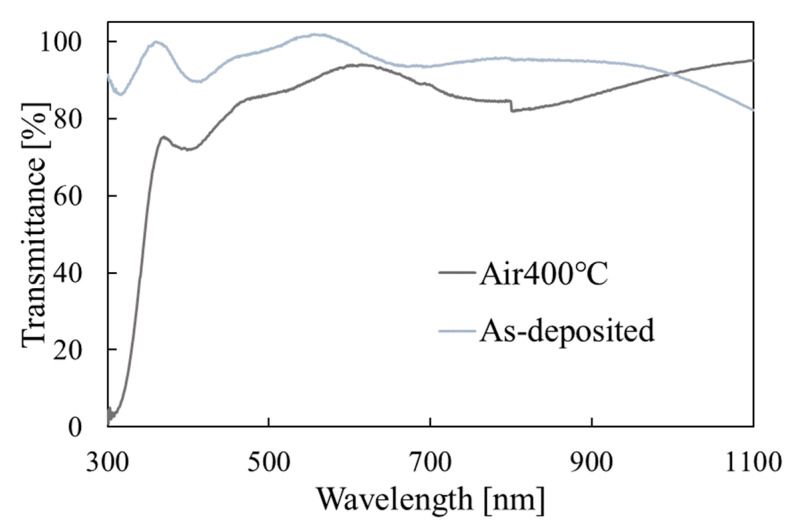
Optical transmittance measurement results for the films before and after annealing.

The transmittance measurement results before and after annealing are shown in Figure 6. Although the as-deposited Ni(OH)_2_ film has no absorption edge in the range of the figure, the annealed NiO film has an absorption edge of around 360 nm. Thus, the change from an insulator to a semi-conductor due to annealing was confirmed. Since NiO has a direct bandgap [1], the bandgap was calculated from the plot of (αhν)^2^ vs. hν, where α is the absorption coefficient and hν is the photon energy. The calculated bandgap is 3.4 eV, which is in the range reported in ref. [1].

**Figure 7:**
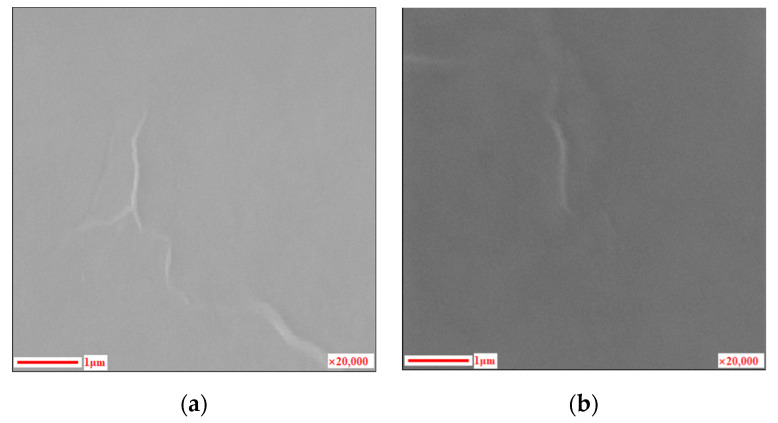
SEM images of films: (**a**) as-deposited; (**b**) annealed at 400 °C for 1 h.

Figure 7 shows the SEM images of the thin film. Grain images were not observed on the surfaces, and the film morphology did not change significantly before and after annealing.

**Figure 8:**
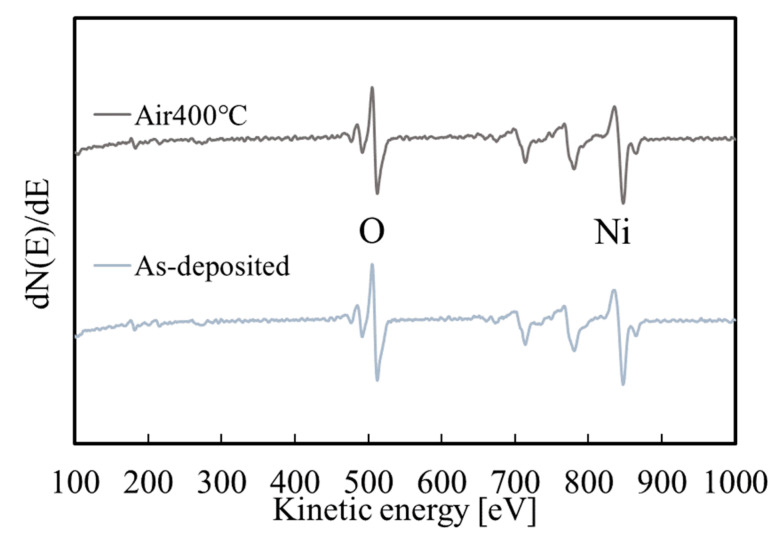
AES measurement results for the films before and after annealing.

The AES measurement results are shown in Figure 8. Argon ion sputtering was performed for 10 s before the measurement. Only Ni and O were detected before and after annealing. Thus, the other elements in the deposition solution (Na, N) were mostly removed by the rinsing process. The composition ratios O/Ni of the thin film before and after annealing are 1.23 and 1.10, respectively; the oxygen content in the thin film decreased as the thin film changed from Ni(OH)_2_ to NiO. The argon ion sputtering before the measurement would result in the decomposition of the hydroxide. Thus, the O/Ni composition ratio before annealing was lower than that expected for Ni(OH)_2_. Similar results have been reported for cobalt hydroxide [44].

**Figure 9:**
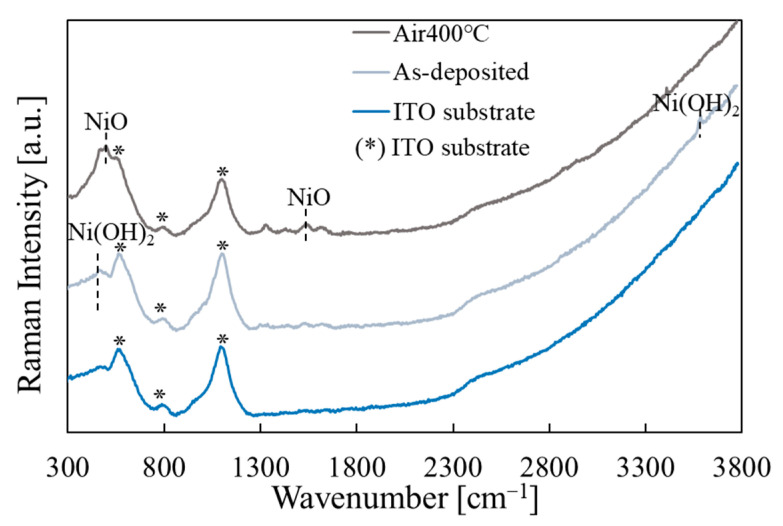
Raman measurement results for the films before and after annealing. For comparison, the spectrum for the ITO substrate is also shown.

Figure 9 shows the Raman measurement results before and after annealing. The peaks of Ni(OH)_2_ and ITO (substrate) were observed before annealing [47], and the peaks of NiO and ITO (substrate) were observed after annealing [48]. Thus, it was found that the Ni(OH)_2_ precursor film was successfully fabricated by DDD, and the film was converted to NiO by annealing.

**Figure 10:**
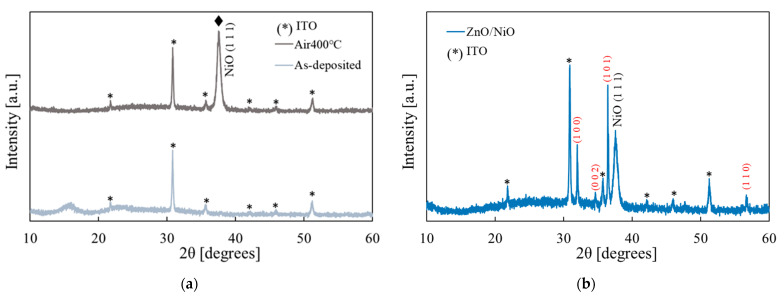
XRD measurement results: (**a**) the Ni(OH)_2_ and NiO films (before and after annealing); (**b**) the ZnO/NiO heterojunction after annealing (the red indices are for ZnO).

The XRD measurement was performed on the films before and after annealing, and the results are shown in Figure 10a. Only the peaks of ITO were observed for the sample before annealing, whereas the peak corresponding to NiO (1 1 1) was also observed after annealing (JCPDS 04-0835). Thus the annealed film was polycrystalline with clear (1 1 1) preferential orientation. Similar results have been reported for the films deposited by the ECD method [37], while the (2 0 0) peak was also observed for NiO films fabricated by other methods [31,49,50].

**Figure 11:**
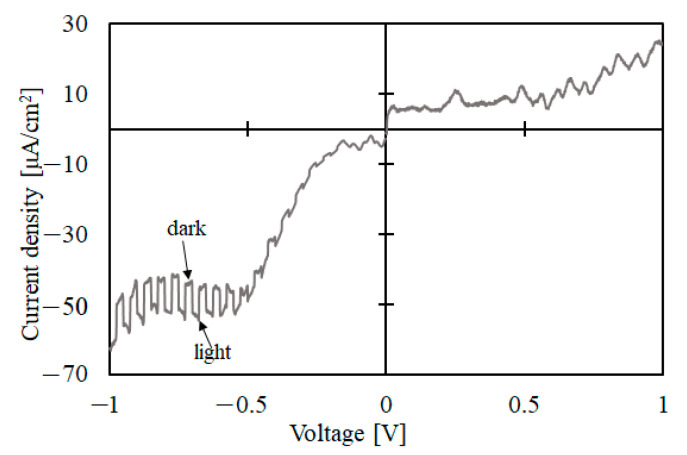
PEC measurement results of the annealed NiO film.

The PEC measurement was performed on the annealed film. The PEC measurement reveals the photo current due to the minority carriers of the semiconductor. The minority carriers of a p-type semiconductor are electrons, and, thus, the photo response will be observed in the negative potential sweep. Conversely, n-type semiconductors respond in the positive potential sweep. The results are shown in Figure 11. The p-type photo response was observed for the annealed thin films, and, therefore, we successfully fabricated p-type NiO thin films by DDD. The photo response was not observed for the Ni(OH)_2_ precursors.

### 3.3. ZnO/NiO Heterojunction

**Figure 12:**
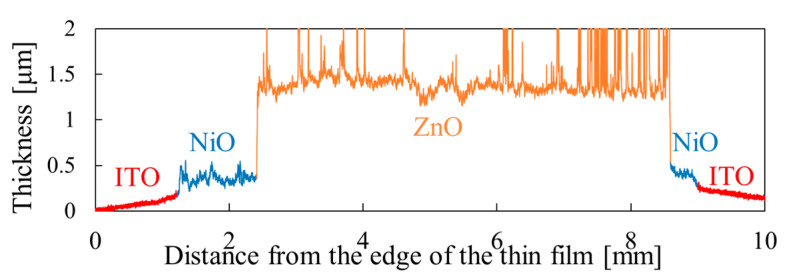
Thickness profile measurement results for the ZnO/NiO heterojunction.

Based on the p-type NiO thin film thus prepared, we fabricated a heterojunction with n-type ZnO and measured the optical and electrical properties. Figure 12 shows the thickness measurement result of the heterojunction. The thickness of NiO was approximately 0.2 μm, and the thickness of ZnO prepared by ECD was approximately 0.9 μm.

**Figure 13:**
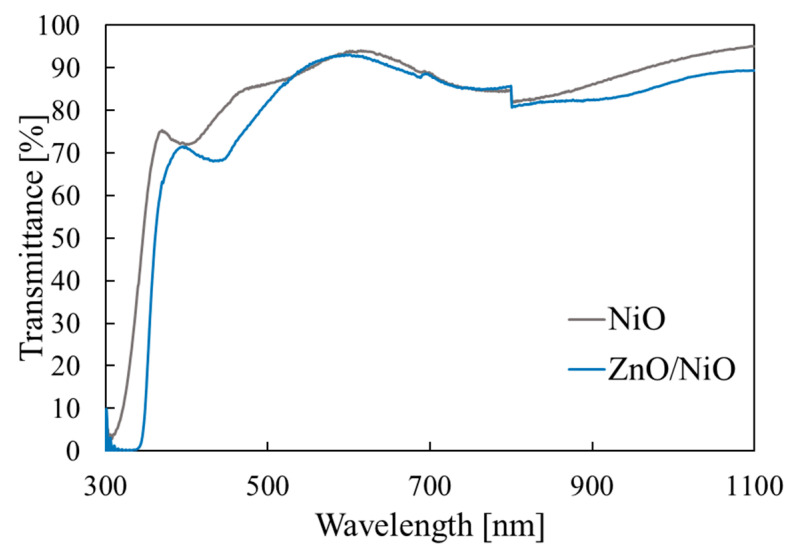
Optical transmittance measurement results for the ZnO/NiO heterojunction.

Figure 13 shows the transmittance of the ZnO/NiO heterojunction and that of the NiO single layer. ZnO has a smaller bandgap than that of NiO, and, thus, the absorption edge in the UV region was red-shifted compared with the spectrum for NiO. The absorption-edge energy of the heterojunction was 3.3 eV, consistent with the literature value for ZnO [46]. The transmittance was more than 70% in the visible range.

The XRD results of the ZnO/NiO heterojunction is shown in Figure 10b. In addition to the peaks of NiO and ITO, the ZnO peaks were also observed (their indices are noted in red in the figure according to JCPDS 36-1451). Thus, the polycrystalline ZnO/NiO heterojunction was successfully prepared.

**Figure 14:**
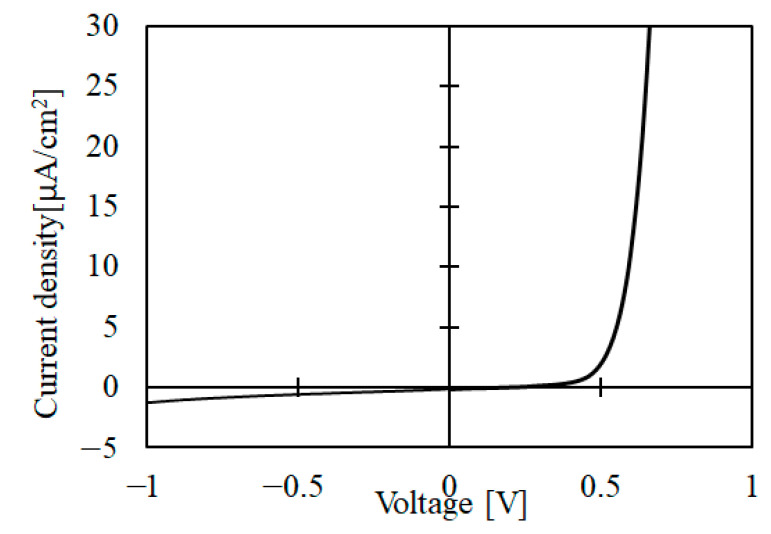
J-V measurement results of the ZnO/NiO heterojunction.

Figure 14 shows the J-V characteristics of the ZnO/NiO heterojunction. It can be seen that the ZnO/NiO heterojunction exhibits a rectification performance. We repeated the fabrication of the heterojunction, and the rectification properties were observed reproducibly.

The successful fabrication of p-type NiO thin films and ZnO/NiO heterojunction demonstrates that the DDD method will be used for the fabrication of NiO-based devices. As mentioned in the introduction, the ZnO/NiO heterojunction has been considered promising for transparent solar cells. Since DDD is so simple, NiO films can be fabricated at a low cost and the solar cell fabrication cost can be reduced. The shortcoming of DDD seems to be the fact that, with an increasing thickness, the thickness uniformity is deteriorated, as discussed in 3.1. Thus, DDD may not be suitable for application where a thick NiO layer is needed. For the ZnO/NiO solar cell application, the NiO layer can be thin since light is mainly absorbed by ZnO, which has a smaller bandgap. A large-area film could be deposited by dropping and spreading the solution from multiple points on the substrate. Thus, DDD would be applicable for the ZnO/NiO solar cells.

As noted in the introduction, the fabrication of ZnO/NiO heterostructure diodes by other chemical solution techniques has been reported [4,10,11,12,13]. The leakage current of the ZnO/NiO cell fabricated in this work seems comparable to those reported previously (of the order of 1 µA/cm^2^). In some of those previous works, photovoltaic properties were observed, but the output voltage was small, of the order of 10 mV [4,13]. In future studies, we will fabricate a ZnO/NiO solar cell based on DDD-deposited NiO.

## 4. Conclusions

Ni(OH)_2_ precursors were prepared by the novel simple technique DDD using an aqueous solution containing Ni(NO_3_)_2_ and NaOH, and converted to NiO thin films by annealing. The films were transparent in the visible range. The XRD and Raman peaks of NiO were observed for the annealed films. The results of the PEC measurement showed that the NiO film was p-type and photoconductive. The ZnO/NiO heterojunction was fabricated with ZnO deposited by ECD, and rectification was observed. Thus, NiO films fabricated by DDD can be used for various electronic and electrochemical applications.

## Data Availability

The data is contained within the article.
